# The *in vivo* study of cardiac mechano-electric and mechano-mechanical coupling during heart development in zebrafish

**DOI:** 10.3389/fphys.2023.1086050

**Published:** 2023-03-16

**Authors:** Jonathan S. Baillie, Alex Gendernalik, Deborah M. Garrity, David Bark, T. Alexander Quinn

**Affiliations:** ^1^ Physiology & Biophysics, Dalhousie University, Halifax, NS, Canada; ^2^ Biomedical Engineering, Colorado State University, Fort Collins, CO, United States; ^3^ Biology, Colorado State University, Fort Collins, CO, United States; ^4^ Mechanical Engineering, Colorado State University, Fort Collins, CO, United States; ^5^ Department of Pediatrics, Washington University in St. Louis, St. Louis, MO, United States; ^6^ Biomedical Engineering, Dalhousie University, Halifax, NS, Canada

**Keywords:** Bainbridge effect, cardiac output, end-diastolic area, Frank-Starling mechanism, heart rate, stretch, stroke volume

## Abstract

In the adult heart, acute adaptation of electrical and mechanical activity to changes in mechanical load occurs *via* feedback processes known as “mechano-electric coupling” and “mechano-mechanical coupling.” Whether this occurs during cardiac development is ill-defined, as acutely altering the heart’s mechanical load while measuring functional responses in traditional experimental models is difficult, as embryogenesis occurs *in utero*, making the heart inaccessible. These limitations can be overcome with zebrafish, as larvae develop in a dish and are nearly transparent, allowing for *in vivo* manipulation and measurement of cardiac structure and function. Here we present a novel approach for the *in vivo* study of mechano-electric and mechano-mechanical coupling in the developing zebrafish heart. This innovative methodology involves acute *in vivo* atrial dilation (i.e., increased atrial preload) in larval zebrafish by injection of a controlled volume into the venous circulation immediately upstream of the heart, combined with optical measurement of the acute electrical (change in heart rate) and mechanical (change in stroke area) response. In proof-of-concept experiments, we applied our new method to 48 h post-fertilisation zebrafish, which revealed differences between the electrical and mechanical response to atrial dilation. In response to an acute increase in atrial preload there is a large increase in atrial stroke area but no change in heart rate, demonstrating that in contrast to the fully developed heart, during early cardiac development mechano-mechanical coupling alone drives the adaptive increase in atrial output. Overall, in this methodological paper we present our new experimental approach for the study of mechano-electric and mechano-mechanical coupling during cardiac development and demonstrate its potential for understanding the essential adaptation of heart function to acute changes in mechanical load.

## 1 Introduction

### 1.1 The importance of cardiac responses to acute changes in mechanical load

The heart is an electrically driven pump that provides the body with the constant supply of blood needed to meet physiological demand. During each heartbeat, muscle cells in the heart (cardiomyocytes) experience a dynamic transformation in dimensions due to varying mechanical forces. During contraction, the active force generated by electrically excited cardiomyocytes must overcome any existing mechanical load and the resistance to blood ejection (afterload) to cause cell shortening, while during relaxation, cardiomyocytes first passively lengthen and then are stretched by the volume of blood filling the heart chambers (preload). Cardiac preload and afterload are constantly changing (for instance during every breath, or change in posture, physical activity, etc.), with acute changes causing rapid alterations in the heart’s electrical and mechanical activity. These rapid changes in cardiac function (rather than slower, central nervous system- or hormonally-mediated alterations) are driven by processes intrinsic to cardiomyocytes, involving feedback from the mechanical environment to their electrical (“mechano-electric coupling,” MEC) and mechanical (“mechano-mechanical coupling,” MMC) activity (Quinn and Kohl, 2016; Quinn and Kohl, 2021). Importantly, this feedback allows for two distinct means of rapidly adjusting cardiac output (CO, the volume of blood pumped by the heart per minute)—which is the product of heart rate (HR, beats per minute) and stroke volume (SV, blood ejected during a single beat)—to acute alterations in venous return (i.e., preload). With increased preload, there is stretch of heart muscle, which will increase CO by increasing HR *via* the “Bainbridge effect” (Quinn and Kohl, 2022) (due to increased automaticity of cells in the heart’s natural pacemaker, the sinoatrial node [SAN] (Quinn and Kohl, 2012)) and contractile force *via* the “Frank-Starling mechanism” (Katz, 2002) (due to changes in myofilament calcium [Ca^2+^] sensitivity, crossbridge- and Ca^2+^-cooperativity, inter-filament spacing, and titin-mediated strain-effects on spatial interrelations of thick and thin filaments in atrial and ventricular cardiomyocytes (Calaghan and White, 1999; de Tombe et al., 2010; Ait-Mou et al., 2016; Neves et al., 2016). The subsequent increase in CO is critical for matching blood ejection to return, to keep the cardiovascular system in balance. Yet, while the existence and importance of these acute stretch responses are well established in the adult heart, their presence and role in the developing heart are ill-defined.

### 1.2 The importance of mechanical load during cardiac development

In the embryo, the heart is one of the first functional organs to develop and is critical for the further development of the whole animal by supplying nutrients to the body and removing waste products. As embryonic growth progresses, the heart transforms from a linear tube into a multi-chambered structure separated by valves for unidirectional blood flow. Critically, this transformation in cardiac morphology is dependent on mechanical changes, manifested as an increase in intracardiac pressure, myocardial strain, and wall shear stress, which result in molecular and intracellular signalling responses that help coordinate cell differentiation and tissue growth. The structural changes that occur during cardiac morphogenesis are critical for the heart’s mechanical function and the effective ejection of blood. For details relating to the critical influence of the heart’s mechanical environment on the development of its structure and mechanical function, we refer the reader to excellent reviews on the subject (Bartman and Hove, 2005; Culver and Dickinson, 2010; Santhanakrishnan and Miller, 2011; Lindsey et al., 2014; Boselli et al., 2015; Happe and Engler, 2016; Duchemin et al., 2019).

The heart’s electrical activity develops along with its structure and mechanical function and is similarly dependent on mechanical factors. This includes establishment of distinct regional differences in electrophysiology, including the atrial and ventricular myocardium (Sedmera et al., 2004; Bressan et al., 2014), the cardiac conduction system (Mikawa and Hurtado, 2007), and the SAN (Opthof, 2007), which is spontaneously active and thus responsible for the initiation of cardiac excitation and the setting of HR (Jansen and Quinn, 2017; Quinn et al., 2021).

Importantly, available evidence suggests that the electrical and mechanical function of the developing heart also respond to acute changes in mechanical load, as in the adult heart. This appears to be important for the pre-neuronal effects of blood pressure on HR (increased blood pressure increases HR in intact chick embryos, prior to cardiac innervation) (Rajala et al., 1976), the coordination of contraction across the heart (when electrical conduction in embryonic chick hearts is prevented by block of gap junctions, the heart continues to beat in a coordinated fashion) (Chiou et al., 2016), and possibly the initiation of the very first heartbeat (as it occurs after fluid pressure build-up in the quiescent heart tube) (Rajala et al., 1977). However, whether rapid functional responses to acutely altered mechanical load (*via* MEC and MMC) are an important adaptive mechanism in the developing heart is unknown.

### 1.3 Zebrafish as an experimental model for the study of cardiac development and function

One reason for our lack of knowledge regarding the importance of MEC and MMC during development are technical and physiological limitations for its study in mammalian models. Acute, controlled *in vivo* alteration of mechanical load, with simultaneous measurements of its functional effects, is difficult in the opaque, *in utero*, mammalian embryo. While recent advances *in vivo* imaging and experimental techniques have provided critical new data regarding the role of mechanics in heart development (Kowalski et al., 2014), information on acute effects of altered load is still lacking.

The zebrafish is a powerful alternative model for studies of development (Kimmel et al., 1995; Hu et al., 2000; Parichy et al., 2009), including the role of mechanics in cardiac morphogenesis (Li et al., 2019; Sidhwani and Yelon, 2019). The most striking advantage of the zebrafish for developmental studies is the ability to easily visualise the developing organs *in vivo* in the transparent, externally growing embryo (Gut et al., 2017). Basic vertebrate organ patterning is conserved in the zebrafish, and zebrafish embryos develop a complete body plan with major organ systems, including a beating heart and major vessels with circulating blood within 48 h post-fertilisation (hpf) (Kimmel et al., 1995). Briefly, cardiac cells of the zebrafish heart begin as two lateral sub-populations, which migrate toward the embryonic midline to form the primitive heart tube. By 24 hpf fusion of the bilateral cardiac precursors is complete and spontaneous waves of excitation and contraction begin, originating from the venous pole of the heart, where the SAN is beginning to form in the ring of tissue between what will become the *sinus venosus* and atrium. By 48 hpf the myocardium has undergone further differentiation and thickening, and the linear tube then “loops” (i.e., bends at the boundary of the atrium and ventricle, such that they come to lie beside each other). This process creates a multi-chambered structure (consisting of the *sinus venosus*, atrium, ventricle, and *bulbus arteriosus*), with slowed conduction in the atrioventricular canal and the formation of one-way valves, allowing for coordinated excitation, contraction, and unidirectional blood flow. At this point the principal features of the heart are present, and the heart continues to grow and undergo further cellular differentiation. For a more in-depth description of zebrafish cardiac development, please see one of the many available comprehensive reviews (Bakkers, 2011; Liu and Stainier, 2012; Brown et al., 2016; Kemmler et al., 2021; Yao et al., 2021).

Importantly, even though the cardiovascular system is one of the first to form during zebrafish development, the embryo obtains adequate oxygen by passive diffusion from water and nutrients from their yolks for the first several days, so that experimental manipulations and mutant phenotypes that severely perturb cardiac function and would be lethal in humans and other mammals can be studied (Stainier et al., 1996). The *in vivo* assessment of heart development in zebrafish embryos is further enhanced by optogenetic tools that allow for the optical measurement and manipulation of cardiac activity in the intact embryo with genetically encoded light-sensitive proteins (Baillie et al., 2021).

There are important factors that also make the zebrafish a powerful model for studies of cardiac function (Stoyek and Quinn, 2018). Genetically, the zebrafish has a fully sequenced genome, which is easily altered using standard genetic techniques at relatively low cost (in terms of time, effort, and money) (Rafferty and Quinn, 2018; Stoyek et al., 2021), and most cardiac genes have a human ortholog with analogous function (although it should be noted that ∼24% of genes have more than one ortholog, due to a duplication of the zebrafish genome, which can confer redundancy in gene function and confound results of genetic manipulations) (Howe et al., 2013). Even though the zebrafish heart is in some ways structurally different than the human heart (e.g., two chambers rather than four) (Hu et al., 2001), functionally it has more comparable HR, action potential morphologies, and electrophysiology to human than rodents (Nemtsas et al., 2010; Vornanen and Hassinen, 2016; Ravens, 2018). However, while Ca^2+^-handling mechanisms are also generally well conserved between zebrafish and humans (Rayani et al., 2018; van Opbergen, et al., 2018), aspects of excitation-contraction coupling do appear to differ. Zebrafish cardiomyocytes may be more dependent on extracellular Ca^2+^ influx (including through T-type Ca^2+^ channels) than intracellular sarcoplasmic reticulum Ca^2+^ release for contraction, due to the apparent low Ca^2+^ sensitivity of their ryanodine receptors (Bovo et al., 2013) and paucity of transverse tubules (Brette et al., 2008) (although others have shown a strong dependence of contractile force on the release of Ca^2+^ from the sarcoplasmic reticulum (Haustein et al., 2015). Zebrafish also have a higher sodium-Ca^2+^ exchanger current than in mammals, such that its reverse-mode has been shown to trigger sarcoplasmic reticulum Ca^2+^ release (Zhang et al., 2011). In regard to the intracardiac (MacDonald et al., 2017) and extracardiac (Stoyek et al., 2016) control of cardiac function in zebrafish, they are similar to human, which is particularly true for the SAN (an important consideration in the context of HR control) (Stoyek et al., 2022) and responses to stretch (which also are more similar to those found in human than rodents) (Werdich et al., 2012; MacDonald et al., 2017).

### 1.4 Studying the effects of mechanical load during cardiac development in zebrafish

Recent innovative studies in zebrafish have provided important novel insights into the role of mechanics in cardiac morphogenesis (Rödel and Abdelilah-Seyfried, 2021). For instance, it has been shown that spatiotemporal variations in wall shear stress coordinate endothelial and valvular growth (Boselli et al., 2017; Bornhorst et al., 2019; Fukui et al., 2021) and trabecular organisation (Lee et al., 2018), and that its genetic (Lee et al., 2018), pharmacological (Yang et al., 2014; Foo et al., 2021), or mechanical (Fukui et al., 2021) disruption results in morphological defects, dependent on mechanosensitive channel-mediated Hippo-YAP-Notch signaling pathways (Lee et al., 2018; Duchemin et al., 2019; Bornhorst et al., 2019; Foo et al., 2021). Studying acute effects of mechanics on cardiac function in development, however, is much more difficult (Watanabe et al., 2016).

In this report we present a novel experimental approach established for the *in vivo* study of the effects of acute alterations in preload on the electrical and mechanical activity of the larval zebrafish heart—to assess the presence of MEC- and MMC-mediated responses during early cardiac development—and present a proof-of-concept study assessing functional changes in the atrium at 48 hpf.

## 2 Methodological development

### 2.1 A novel *in vivo* approach for acutely increasing cardiac preload in larval zebrafish

Our new approach for studying MEC and MMC in larval zebrafish builds off recent work using a hydrostatic pressurisation technique designed to mechanically load the atrium *in vivo*, for the quantification of passive mechanical properties of the myocardium during cardiac development (Gendernalik et al., 2021). Similar experiments have been performed previously in chick hearts (Tobita et al., 2002), however that work was limited to *ex vivo* studies using explanted hearts. We have chosen to focus on the functional effects in the atrium (rather than the ventricle), as the SAN is located at the border between the *sinus venosus* and atrium, so will be affected by atrial (but not ventricular) mechanical load, and is the cardiac tissue that most clearly displays an MEC response (the Bainbridge effect). Further, at early stages of zebrafish development the atrium provides much of the work required for circulation (Bark et al., 2017), so the MMC response (the Frank-Starling mechanism) is expected to be greatest in that chamber. Finally, in preliminary investigations it was found that the injection of fluid into the venous circulation of larval zebrafish immediately upstream of the heart causes greater atrial than ventricular dilation, as once fluid enters the ventricle forward flow is already increased, due to atrial MEC and MMC responses (to effectively dilate the ventricle requires an even greater injection of fluid, which causes over-distention of the atrium and destabilises heart rhythm).

Here we describe the evolution of our approach from the previous hydrostatic pressurisation technique to the current electronic flow control system and demonstrate its use by investigating MEC and MMC responses in the atrium of 48 hpf zebrafish larvae by determining the effects of increased preload on its electrical activity (assessed by changes in HR, as in sinus rhythm HR is determined solely by SAN) and mechanical function (assessed by changes in atrial stroke area).

### 2.2 Previously employed technique: Hydrostatic pressurisation

In previous *in vivo* studies examining the passive mechanical properties of the developing zebrafish atrium, changes in mechanical load were applied by a hydrostatic pressurisation technique (Gendernalik et al., 2021). Briefly, this consisted of a saline-filled fluid column connected to a micro-cannula (with a 10–15 μm outer diameter) and a syringe pump to control of the level of fluid in the column and produce specified pressures. Zebrafish larvae were embedded ventral side up in low-melt agarose (for physical immobilisation) containing 2,3-butanedione monoxime (to prevent contraction of the heart) under an upright fluorescence microscope, and the micro-cannula was advanced with a micromanipulator to puncture the common cardinal vein, downstream of the junction of the sub-cardinal veins and slightly upstream of where it drains into the *sinus venosus*. This placement ensured that the volume injected out of the micro-cannula entered the atrium, while avoiding direct mechanical interaction of the cannula with the SAN. Importantly, cannulation alone did not affect the size or morphology of the heart or initiate an electrical or mechanical response. After the micro-cannula was inserted, pressure was incrementally increased, with brightfield images of the heart collected through the upright microscope. This approach allowed for the application of low levels of sustained static pressure to the larval zebrafish atrium and visualisation of the resulting deformation, which was used to obtain pressure-stretch relationships for constitutive models of the myocardium and estimates of tissue stiffness during development. For further details and specifics about this system and its application, please see the report by (Gendernalik et al., 2021).

While the system is elegant in its simplicity and ease of use, the resolution of changes in applied pressure is limited and it does not allow for the rapid application of mechanical loading needed for the assessment of acute MEC and MMC responses in the beating heart.

### 2.3 Updated technique: Electronic pressure control

To allow for rapid injection of small volumes to dilate the heart and assess acute MEC and MMC responses, we modified the previous pressurisation approach to one using a high-resolution electronic pressure control system. For this, the hydrostatic column was replaced by a microfluidic pneumatic device that pressurised a 15 mL solution-containing conical tube (LU-FEZ-2000, Fluigent, Paris, France; range = 0–2000 mbar, resolution = 600 μbar, step size = 0.03% of maximum pressure, stability = 0.1%, response time = 30 ms), to generate controlled flows that are dependent primarily on system resistance and fluid viscosity. The conical tube outlet was connected to the micro-cannula, so that the pressure resulted in the outflow of solution. Pressurisation was controlled by computer (connected *via* a LINK module, Fluigent), using commercial software designed for the pressure control device (OxyGEN, Fluigent), to allow for custom time-based pressurisation protocols and outlet pressure recordings.

While this updated approach allowed for more rapid and precise changes in cannula outlet pressure, the rate of outflow from the micro-cannula varied between each preparation, since outflow is a function of system resistance, which varies due to differences in cannula tip diameter, the nature of the cannula puncture, and subject-specific anatomy. As a result, the injected volume and resulting atrial dilation from a given pressure were difficult to predict and varied greatly from heart to heart.

### 2.4 Current technique: Electronic flow control

**FIGURE 1:**
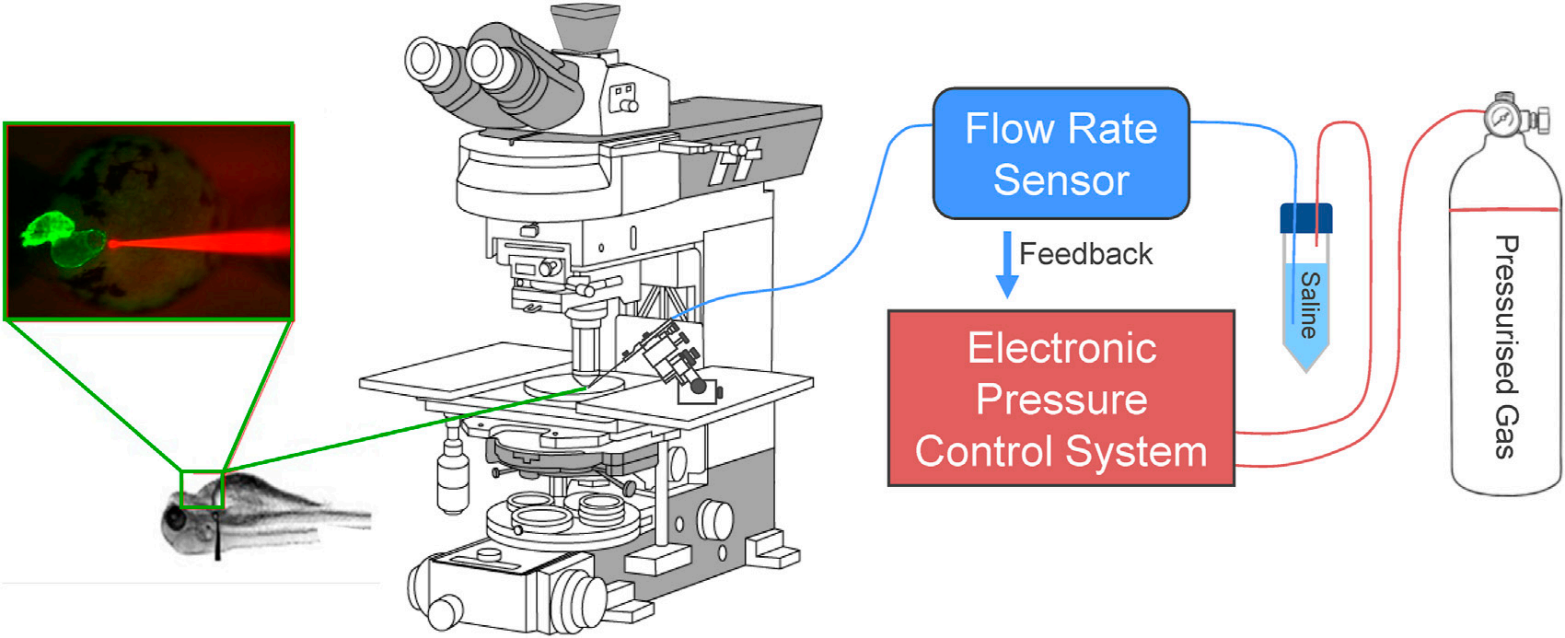
Electronic flow control system for acutely increasing cardiac preload in intact larval zebrafish. Outlet pressure of a pressurised gas cylinder is modulated by a computer-controlled high-resolution electronic pressure control system, which pressurises a conical tube filled with saline solution. Flow rate from the tube is measured by a bidirectional flow rate sensor and maintained at a set value by adjusting pressure. A micro-cannula is connected to the flow outlet, advanced with a micromanipulator under an upright fluorescence microscope to puncture the common cardinal vein (slightly upstream of where it drains into the sinus venosus), and used to inject volume into the heart. Effects of volume loading are visualised through the microscope with an industrial camera.

**FIGURE 2:**
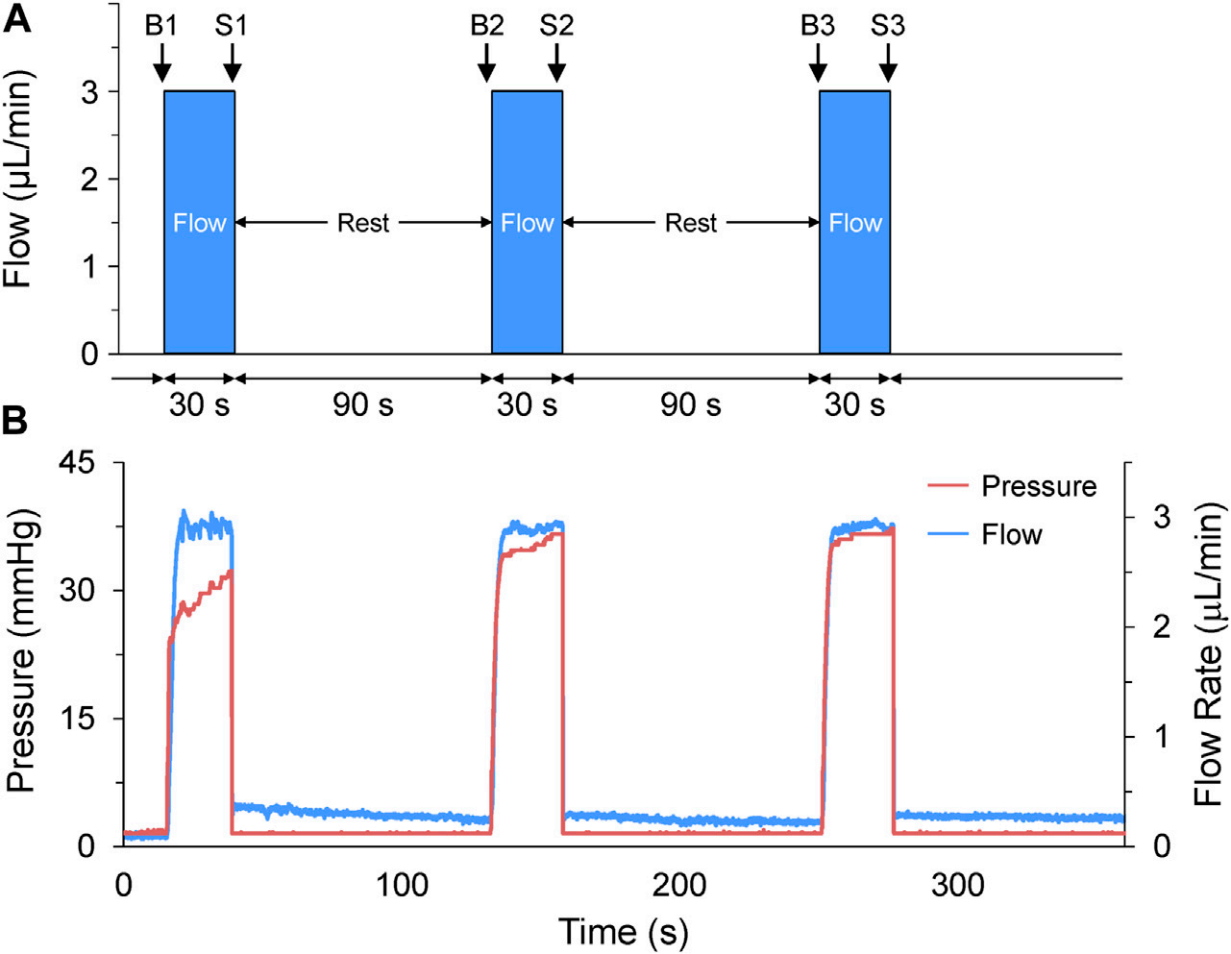
Acute volume loading protocol. **(A)** Acute volume loading involved three 30 s periods of 3 μL/min saline injection through the micro-cannula, with a 90 s rest period between each, and measurements taken immediately before (B1-B3) and at the end of load application (S1-S3). **(B)** The pressure and flow during loading were monitored and recorded by the pressure-flow control software.

**FIGURE 3:**
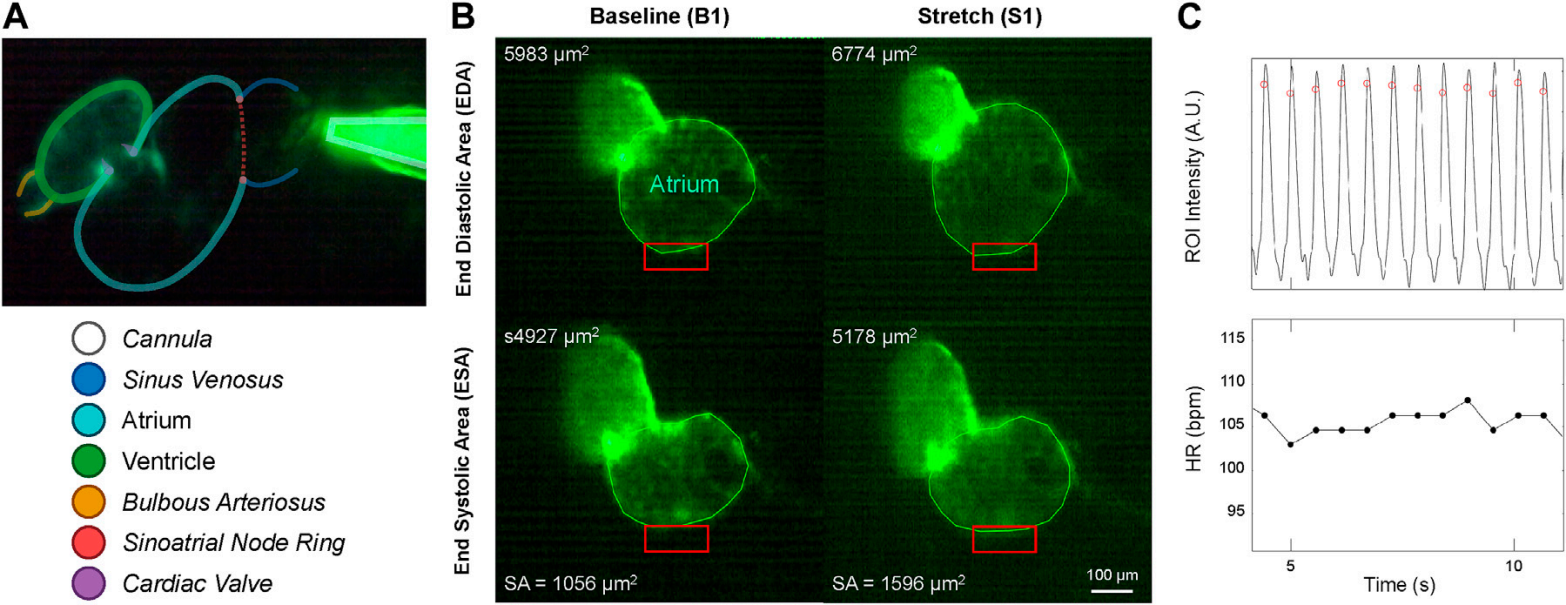
Micro-cannula placement and measurement of atrial dilation and functional effects. **(A)** *In vivo* image of the 48 hpf zebrafish heart expressing eGFP (*Tg(myl7:eGFP*) and the micro-cannula filled with fluorescein, showing the relative position of the cannula to various cardiac regions. **(B)** Videos of heart during atrial dilation were analysed in Matlab to calculate function parameters at baseline immediately before (B1-B3) and at the end of load application (S1-S3). Atrial end-diastolic (EDA) and end-systolic (ESA) area were measured by tracing the area of the atria at the end of filling and the end of ejection, respectively, from which stroke area (SA) was calculated as EDA - ESA. **(C)** HR was measured from the time between peaks of filtered image intensity signals, acquired by averaging intensity within a region of interest placed over the atrial wall (red boxes in **B**).

To rectify the variability in injected volume and atrial dilation, we moved to a controlled flow system. In this configuration, the addition of a bidirectional flow rate sensor inline with the micro-cannula outlet allows for the control and monitoring of flow rate between 0–80 μL/min with an accuracy of 5% (FLU-M-D, Fluigent), by automatic pressure adjustment (Figure 1). Like the pressure control system, flow is controlled by computer, allowing for custom protocols and the recording of pressure and flow (Figure 2). During volume injection, videos of the heart tagged with a cardiac-specific fluorescent marker are taken through an upright microscope, which allows for measurement of HR and atrial dimensions (Figure 3).

Below, we demonstrate the use of this novel system in a proof-of-concept study investigating MEC and MMC responses to increased preload in the atrium of 48 hpf zebrafish larvae.

## 3 Proof-of-concept application

### 3.1 Assessing the adaptative response of atrial function to an acute increase in preload during early cardiac development

All experimental procedures were approved by the Dalhousie University Committee for Laboratory Animals and followed the guidelines of the Canadian Council on Animal Care. Details of experimental protocols have been reported following the Minimum Information about a Cardiac Electrophysiology Experiment (MICEE) reporting standard (Quinn et al., 2011).

### 3.2 Zebrafish larvae preparation

Zebrafish were bred and raised by the Faculty of Medicine Zebrafish Core Facility at Dalhousie University. 48 hpf zebrafish with a point mutation in the *mitfa* and *roy* genes, resulting in a lack of melanocytes and iridophores that makes them mostly transparent (a common background strain known as *casper* (White et al., 2008)), genetically expressing a fluorescent marker (eGFP) specifically in cardiomyocytes (driven by the heart-specific myosin light chain 7 [*myl7*] promoter, to make the heart easier to visualise) were used for this study (*Tg*(*myl7*:*eGFP*) on a *casper* background). Animals were anesthetised in 0.35 mM (90 mg/L) Tris-buffered tricaine solution (pH 7.4; Tris: BP152, Fisher Scientific, Waltham, MA; tricaine: MS-222, Sigma-Aldrich, St. Louis, MO) for 5 minutes (which provides adequate anesthesia, while causing no effects on HR or the heart’s mechanical function), transferred to a glass depression slide (CSTK01, United Scientific Supplies, Libertyville, IL), and embedded ventral side up in 1.5% low-melting agarose (IB70051, IBI Scientific, Dubuque, IA) in HEPES-buffered saline solution (in mM: NaCl 142, KCl 4.7, MgCl_2_ 1, CaCl_2_ 1.8, Glucose 10, HEPES 10) with Tris-buffered tricaine. All experiments were performed at a controlled room temperature of 21°C (which is below the “physiological” temperature of 28°C at which zebrafish are maintained in our zebrafish CORE facility, but not thought to affect the presence of cardiac stretch responses). Zebrafish with an initial HR less than 60 beats per minute were not used for experimentation.

### 3.3 Cannulation of the venous circulation

Borosilicate glass capillaries (100 mm, 1 mm outer/0.58 mm inner diameter; 1B100-4, World Precision Instruments, Sarasota, FL) were shaped using a micropipette puller (P-97, Sutter Instruments, Novato, CA) into cannulas with a tip diameter of ∼4 μm. These micro-cannulas were coupled to the high-resolution electronic flow control system described above and secured to a three-axis micro-manipulator (MM-3, Narishige, Amityville, NY) on the stage of an upright fluorescence microscope (Eclipse 80i, Nikon, Tokyo, Japan) with a 10×, 0.30 NA air objective (Plan Fluor, Nikon). As described above, the micro-cannula was inserted through the agarose and the skin of the larval zebrafish and into the venous circulatory system at the common cardinal vein, downstream of the sub-cardinal veins and upstream of the *sinus venosus* (Figure 3).

### 3.4 Volume injection for controlled atrial dilation

Volume loading protocols were developed in the flow control software (OxyGEN, Fluigent), by defining the rate and duration of flow of HEPES-buffered saline solution (in mM: NaCl 142, KCl 4.7, MgCl_2_ 1, CaCl_2_ 1.8, Glucose 10, HEPES 10) out of the micro-cannula. The combination of flow rate and duration were optimised for 48 hpf larval zebrafish by co-varying the parameters in preliminary experiments (flow rates of 1–5 mL/min and durations of 5–60 s) to determine the combination that resulted on average in a ∼25% increase in atrial end-diastolic area (EDA, which is the degree of stretch that gives the most robust HR response in SAN isolated from adult zebrafish (MacDonald et al., 2017)), and no more than a 50% increase (as preliminary experiments showed that much like stretch of the adult SAN, dilation of greater than 50% destabilised rhythm). The optimal combination was found to be a cannula flow of 3 μL/min for 30 s. It should be noted that a ∼25% increase in atrial EDA (which, assuming the 48 hpf zebrafish atrium is a sphere, equates to a ∼40% increase in atrial volume) was chosen to generate a robust functional response to assess the presence of MEC and MMC effects in the developing heart, so does not necessarily relate to the change in atrial preload that might occur under normal physiological conditions in the embryonic heart. The volume injection was applied three times, with a 90 s rest period between each loading phase (Figure 2A), as 90 s was determined in preliminary experiments to be a sufficient amount of time to allow for atrial EDA to return to baseline. Pressure and flow during the loading protocol were monitored and recorded using the OxyGEN (Fluigent) software (Figure 2B).

### 3.5 Measuring functional effects of acute atrial dilation

Videos of the beating heart were acquired with a colour industrial CMOS camera (DFK 33UP-1300, The Imaging Source, Charlotte, NC) at 60 frames per second on the upright microscope by exciting the eGFP with a super high pressure mercury lamp (HB-10101AF, Nikon) passed through a 480/40 nm filter and a 506 nm dichroic mirror, with emission collected through a 535/50 nm filter (Chroma, Bellows Falls, VT). Videos were analysed using custom routines in Matlab (MathWorks, Natick, USA) to calculate functional parameters immediately before and at the end of each application of atrial preload. Atrial EDA and end-systolic area (ESA) were measured by tracing the area of the atrium at the end of filling and the end of ejection, respectively. Atrial stroke area (SA) was calculated as EDA—ESA, and an area-based index of atrial CO (CO_A_) was calculated as HR × SA (Figure 3A). HR was measured from the time between peaks of a temporally filtered (60 Hz) image intensity signal, acquired by averaging intensity within a region of interest placed over the atrial wall (Figure 3B).

### 3.6 Statistical analysis

Values are reported as mean ± SEM. Statistical analysis was performed in Prism (GraphPad, San Diego, CA). Group means were compared either by paired two-tailed Student’s T-tests or repeated measures or one-way ANOVA, with Tukey or Dunnet *post hoc* tests for pairwise comparisons, as appropriate. Significance was indicated by *p* < 0.05.

## 4 Results

### 4.1 Acute atrial dilation with volume loading in 48 hpf zebrafish larvae

**FIGURE 4:**
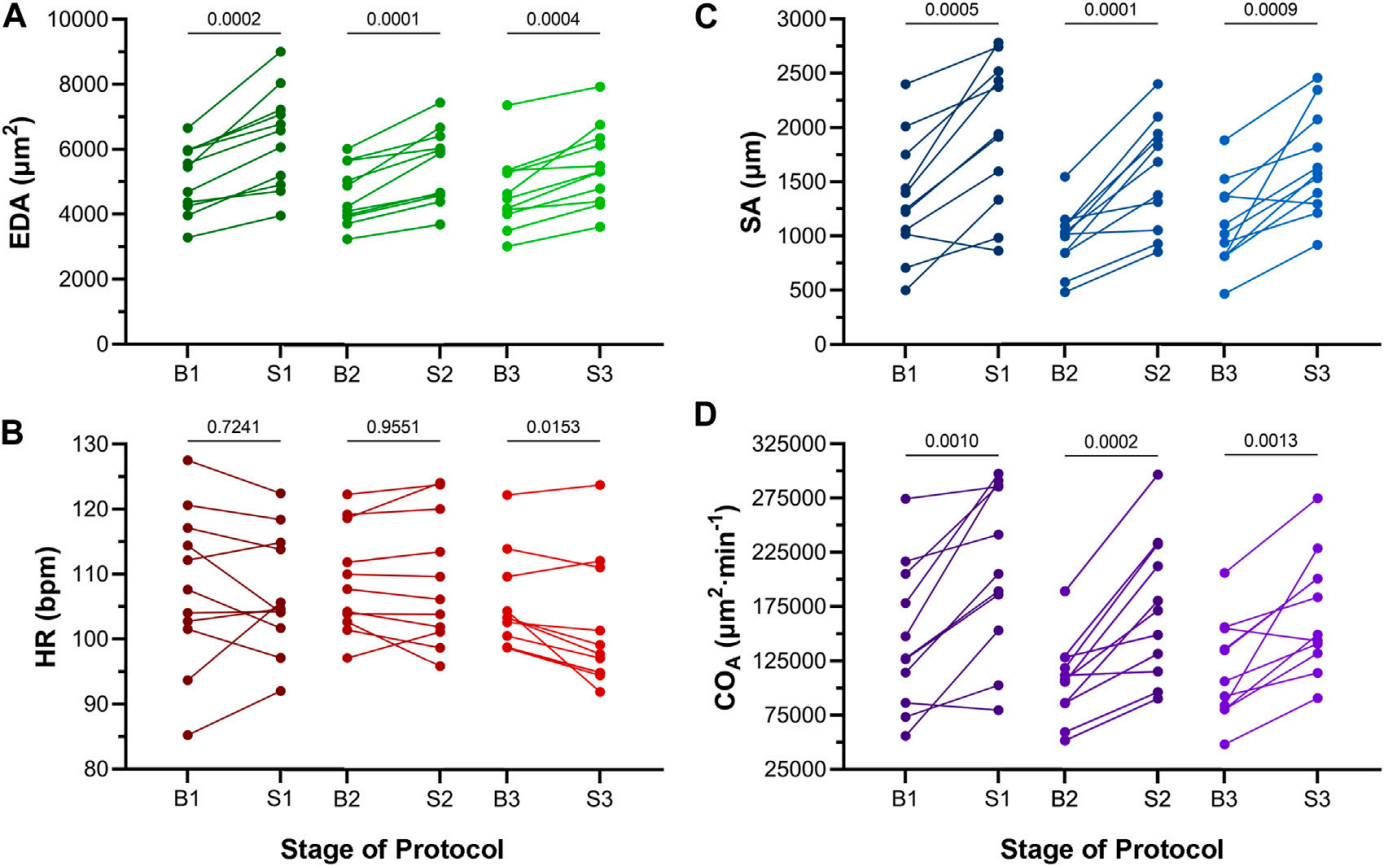
Effects of atrial dilation on functional parameters in 48 hpf zebrafish larvae. **(A)** End-diastolic area (EDA), **(B)** heart rate (HR), **(C)** stroke area (SA), and **(D)** area cardiac output (CO_A_ = HR × SA) immediately before (B1-B3) and at the end of load application (S1-S3). Mean values before and during each stretch were compared by paired two-tailed Student’s T-tests. Significance was indicated by *p* < 0.05. *n* = 11 larvae.

**FIGURE 5:**
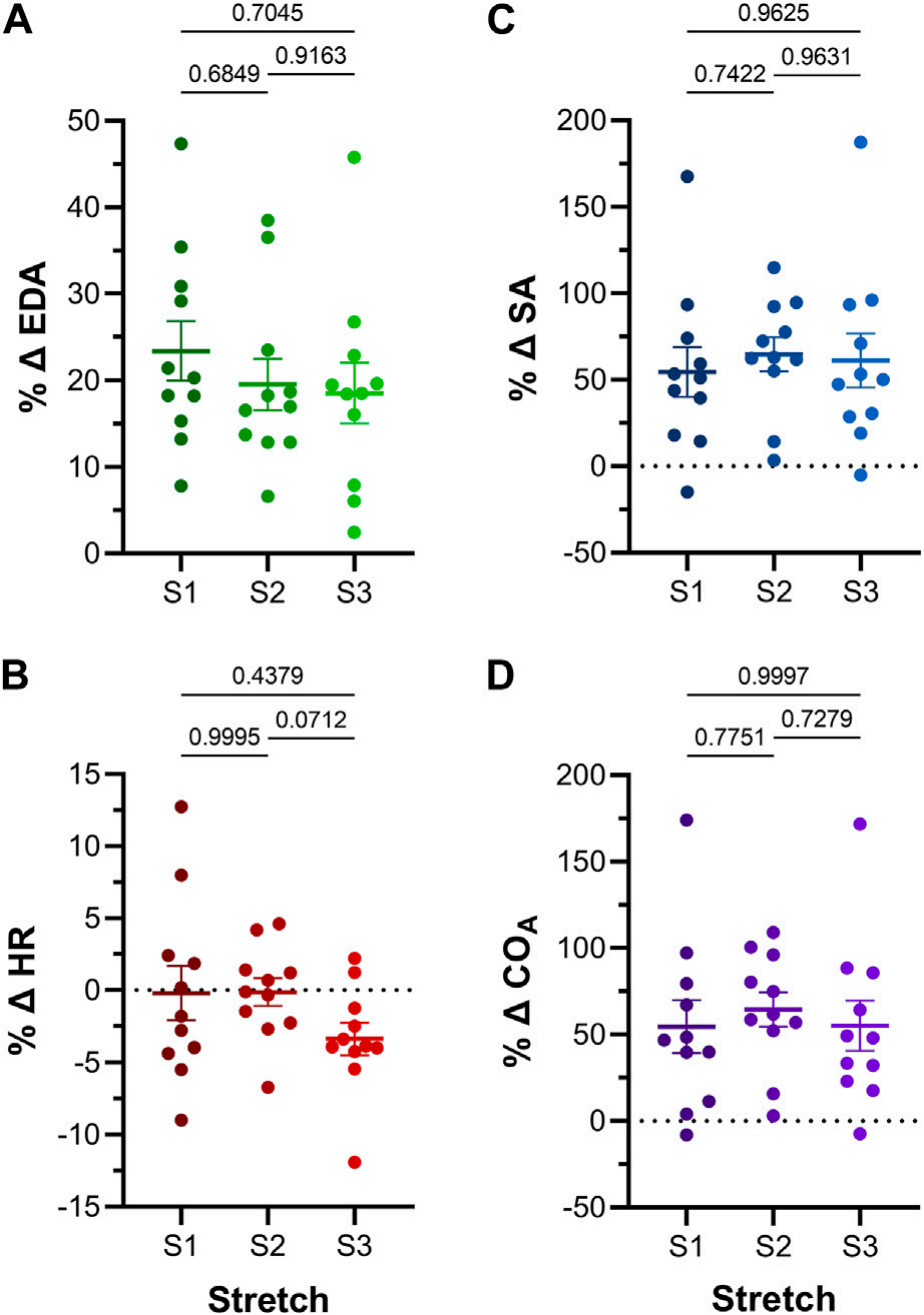
Effect of repeated atrial preload application on functional parameters in 48hpf zebrafish larvae. Percentage change (%Δ) of atrial **(A)** end-diastolic area (EDA), **(B)** heart rate (HR), **(C)** stroke area (SA), and **(D)** output (CO_A_) for each period of load application (S1, S2, S3). Average values presented as mean ± SEM. Means for each loading period were compared by repeated measures ANOVA, with Tukey *post hoc* tests for pairwise comparisons. *n* = 11 larvae.

**FIGURE 6:**
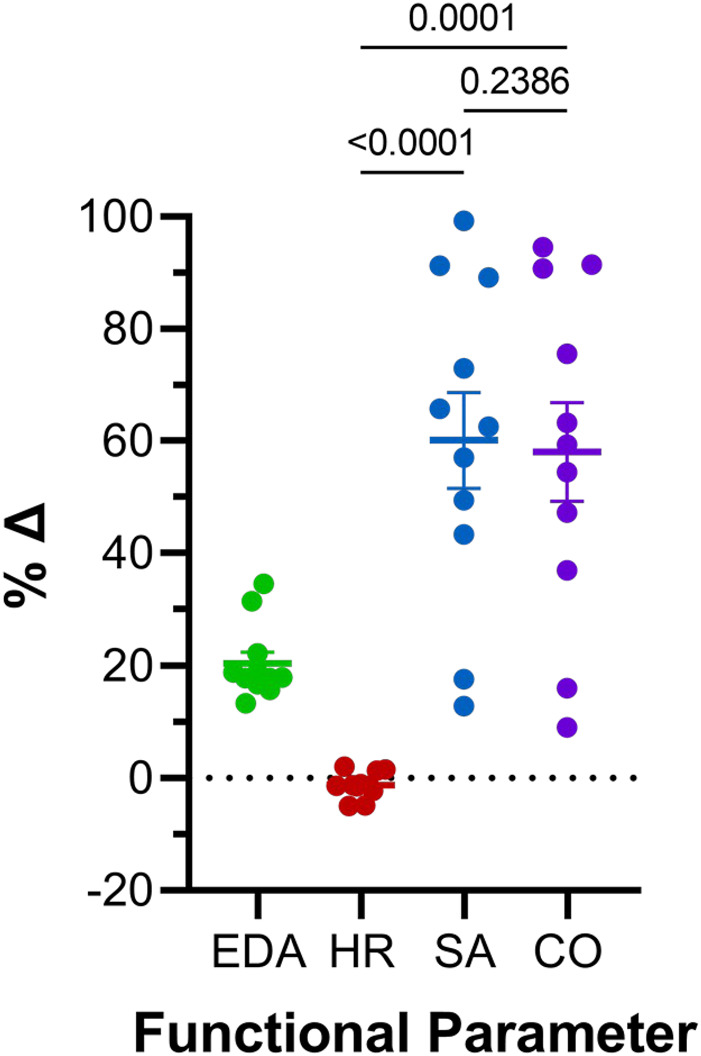
Comparison of effects of atrial preload application on functional parameters in 48 hpf zebrafish larvae. Percentage change (%Δ) of end-diastolic area (EDA), heart rate (HR), stroke area (SA), and area cardiac output (CO_A_) averaged over all periods of load application. Average values presented as mean ± SEM. Means for each measured parameter were compared by one-way ANOVA, with Tukey *post hoc* tests for pairwise comparisons. *n* = 11 larvae.

Atrial EDA was acutely and transiently increased three times by the controlled injection of saline into the common cardinal vein (3 μL/min for 30 s). Figure 4A shows the measured EDA immediately before (B1-B3) and at the end (S1-S3) of each loading period—with each injection of saline there was a significant increase in atrial EDA. Importantly, during the rest period between each saline injection, EDA returned to baseline (B1 vs*.* B2: *p* = 0.063, B2 vs*.* B3: *p* = 0.896, B1 vs*.* B3: *p* = 0.101, by Tukey *post hoc* tests with repeated measures ANOVA), indicating that there was no residual increase in atrial preload with successive volume injections. The amount of atrial dilation with each injection was also similar (Figure 5A), indicating a consistent effect on atrial preload, and justifying averaging across all injections (a 20.5% ± 2.0% increase in atrial EDA; Figure 6).

### 4.2 Electrical and mechanical response to acutely increased atrial preload

The effect of the increase in EDA on the electrical and mechanical function of the atrium was assessed by comparing HR and SA before and during each volume injection. Figures 4B, C show the measured HR and SA before and at the end of each loading period. The first two periods of acute loading had no effect on HR, while the third caused a small decrease. For SA, there was a significant increase with each loading period. For both HR and SA there was no difference between values immediately before the application of load, indicating a return to baseline during the rest period for each (HR – B1 vs*.* B2: *p* = 0.929, B2 vs*.* B3: *p* = 0.205, B1 vs*.* B3: *p* = 0.722; SA – B1 vs*.* B2: *p* = 0.057, B2 vs*.* B3: *p* = 0.409, B1 vs*.* B3: *p* = 0.342). As for EDA, there was no difference in the change in SA with each load application (Figure 5C), and the small decrease in HR during the third loading period (−3.4% ± 1.1%) was not significantly different than the first two periods, which showed no change (Figure 5B). When averaged across all volume injections, the application of load resulted in a 60.1% ± 8.5% increase in SA (Figure 6).

### 4.3 Effect of altered electrical and mechanical function on atrial output

With an increase in venous return to the heart there must be a concomitant increase in CO—to match cardiac outflow to inflow and maintain hemodynamic balance—which can occur through an increase in HR, SA, or both. Figure 4D shows the effect of the increase in atrial EDA on the area-based index of atrial CO (CO_A_). With each dilation of the atrium, there was a significant increase in CO_A_, which during the rest period returned to the baseline value (B1 vs*.* B2: *p* = 0.112, B2 vs*.* B3: *p* = 0.631, B1 vs*.* B3: *p* = 0.310). There was no difference in the change of CO_A_ with each application of load (Figure 5D), which when averaged across all volume injections was 58.1% ± 8.8% (Figure 6). This increase in CO_A_ was driven solely by an increase in SA, supported by the lack of difference in the percentage change of CO_A_ vs*.* SA, and a difference in the change of both compared to the lack of an effect on HR (Figure 6).

## 5 Discussion

Here we have presented a novel method for consistent and repeatable rapid *in vivo* application of mechanical load to the larval zebrafish heart, combined with measurement of its acute effect on electrical and mechanical function as a means to study the emergence of MEC and MMC responses in cardiac development. In a proof-of-concept application, we demonstrated that in 48 hpf zebrafish larvae, an acute increase in atrial preload has little effect on HR, but causes a large increase in atrial SA, such that the concomitant increase in atrial CO_A_ is driven solely by MMC mechanisms. Overall, our results demonstrate the potential of our new methodology for further studies exploring the importance and mechanisms of mechanical control of cardiac function during heart development.

### 5.1 The functional response of the zebrafish atria to an acute increase in preload during early development

With an average acute increase in atrial EDA of ∼20% in 48 hpf zebrafish larvae, we found close to a 60% increase in atrial CO_A_, with a similar increase in atrial SA and little change in HR. This suggests that acute adaptation to changes in preload at early developmental stages in the zebrafish heart are driven solely by MMC mechanisms, which involve a stretch-induced increase in myofilament interactions due to a combination of increased myofilament Ca^2+^ sensitivity, crossbridge- and Ca^2+^-cooperativity, reduced inter-filament spacing, and titin-mediated changes in the spatial arrangement of thick and thin filaments (Calaghan and White, 1999; de Tombe et al., 2010; Ait-Mou et al., 2016; Neves et al., 2016). This is in contrast to the heart of adult zebrafish (MacDonald et al., 2017) and other vertebrates (Quinn and Kohl, 2012), in which there is also a robust rapid increase in HR with stretch of the SAN, such that a combination of both MEC and MMC responses are responsible for the associated increase in CO that ensures the matching of output to venous return (Quinn and Kohl, 2016), suggesting that the mechanisms driving MEC in the zebrafish heart (or at least the SAN) are not yet present at 48 hpf, and emerge later in cardiac development.

Previous studies in embryonic avian and zebrafish hearts, however, have provided evidence suggesting that both the electrical and mechanical function of the heart respond to acute changes in mechanical load during development. Electrically, prior to cardiac innervation in 72 hpf intact chick embryos and isolated embryonic hearts (meaning effects of the neuronally-mediated Baroreceptor reflex on heart function are not yet active), it has been shown that an acute increase in blood pressure results in an increase in HR (Rajala et al., 1976). Mechanically, computational modelling has suggested that mechanical factors may coordinate contraction across the heart, even without electrical excitation (Chiou et al., 2016), which may also drive initiation of the very first heartbeat in the quiescent heart tube of ∼30 hpf chick embryos (Rajala et al., 1977). While the mechanisms responsible for these acute cardiac responses to changes in the heart’s mechanical environment are unknown, they could be driven by MMC or MEC effects. Our data in zebrafish would suggest that MEC mechanisms are not yet present in the heart at early developmental stages, so MMC may be the primary driver of increased output, however the emergence of MEC and MMC mechanisms may differ between species.

In the zebrafish, Werdich et al. demonstrated a transient increase in HR with stretch of the ventricle in hearts isolated from 72 hpf embryos (Werdich et al., 2012). This suggests that MEC mechanisms may emerge between 48 hpf (when we saw no HR response) and 72 hpf (when Werdich et al. observed a response). However, the HR effect seen with ventricular stretch in isolated hearts in the previous study, and its comparison with our *in vivo* results, should be interpreted be with caution. One possibility for the difference in HR response between the two studies—other than the timing of the emergence of MEC mechanisms—could relate to differences between effects seen the isolated vs*. in vivo* heart. There may be additional mechanisms present *in vivo* that oppose the stretch response seen in isolated hearts (resulting in no change in HR), such as the baroreceptor (Bezold-Jarisch) reflex, which reduces HR when arterial blood pressure is increased (Bezold, 1867; Jarisch and Richter, 1939). This neuronally-mediated effect, however, is unlikely to be active in 48 hpf zebrafish embryos, as available evidence indicates that the heart is not yet innervated at that stage of development (Stoyek et al., 2021). A more likely explanation for the difference in the HR response between the two studies relates to the nature of the applied mechanical load. In our study, we increase atrial preload with the injection of fluid into the venous system, which resulted in a 20.5% ± 2.0% increase in atrial EDA (corresponding to approximately a 32% ± 3% increase in atrial volume), while in the study by Werdich et al. they used carbon fibres on either side of the ventricle to apply and increase that dimension by 230% ± 50%. Thus, the stretch applied in the previous study was drastically different than in our study, both in magnitude (230% vs*.* 32%) and in nature (linear cross-chamber tissue stretch vs*.* chamber dilation). There is, however, an even more puzzling aspect of the study by Werdich et al. that should be considered. In their study, stretch was applied to the ventricle, so should not have resulted in a change in HR, as the Bainbridge effect is driven by stretch of the SAN, which in the zebrafish is found in the ring at the border of the *sinus venosus* and atrium. This suggests that the observed increase in HR in fact represents stretch-induced ectopic excitation or automaticity arising from within the ventricle (or possibly the atrioventricular junction).

Overall, our findings in zebrafish and those of others described above warrant further studies regarding the timing of the emergence of cardiac MEC and MMC in the developing heart, and the underlying mechanisms driving their effects, made possible by our novel methodology.

What is already well established is the critical role that mechanical load, including intracardiac pressure, myocardial strain, and wall shear stress, plays in cardiac morphogenesis and the establishment of normal mechanical and electrical heart function during development, by driving molecular signalling involved in the coordination of cell differentiation and tissue growth (Bartman and Hove, 2005; Culver and Dickinson, 2010; Santhanakrishnan and Miller, 2011; Lindsey et al., 2014; Boselli et al., 2015; Happe and Engler, 2016; Duchemin et al., 2019). Some of the critical insights regarding the dependence of cardiac development on mechanics has come from *in vivo* studies in zebrafish larvae, which have shown the importance of spatiotemporal variations in wall shear stress for coordinating endothelial and valvular growth and trabecular organisation (Yang et al., 2014; Boselli et al., 2017; Lee et al., 2018; Bornhorst et al., 2019; Foo et al., 2021; Fukui et al., 2021). Our new methodology for studies in zebrafish is also well suited to allow for experiments aimed at gaining further insights into the critical role of mechanics in the development of cardiac structure and function.

### 5.2 Future applications of novel methodology for controlled *in vivo* application of mechanical load to the larval zebrafish heart

The presented novel methodology for the *in vivo* study of functional effects caused by acute changes in the heart’s mechanical load in zebrafish larvae has the potential to shed new light into the emergence and importance of MEC and MMC in cardiac development. Here we have demonstrated its use to study the effect of an increase in atrial preload in 48 hpf zebrafish, but it is applicable to later stages of development and may be adapted to study effects in the ventricle. Using the largely transparent *casper* zebrafish allows the heart to be clearly visualised *in vivo* until approximately 30 days post fertilisation (at which point the heart is fully formed (Hu et al., 2000)), so future studies may delineate the role of MEC and MMC in the acute adaptation of function to changes in mechanical load throughout the entirety of cardiac development. Moreover, through that period our experimental approach may be combined with fluorescent imaging in transgenic zebrafish with cardiac-specific expression of functional fluorescent probes, such as genetically-expressed voltage (GEVI) or Ca^2+^ (GECI) indicators (Baillie et al., 2021) for the *in vivo* measurement of membrane potential or intracellular Ca^2+^ handling to gain insight into subcellular mechanisms driving responses to acute changes in mechanical load (Baillie et al., 2021). Using the zebrafish also allows for genetic manipulations, to more specifically understand underlying molecular mechanisms (Rafferty and Quinn, 2018; Stoyek et al., 2021). The zebrafish is also an excellent model for cardiac disease (Gut et al., 2017; Echeazarra et al., 2021; González-Rosa, 2022), so that potential contributions of MEC and MMC—and their disruption—to cardiac structural malformations and functional deficits in development may be studied, to better understand congenital heart disease and early cardiac dysfunction. Finally, as imaging technology continues to advance, the above may be enhanced by the use of four-dimensional imaging techniques (Vedula et al., 2017; Sacconi et al., 2022), for a more comprehensive, whole heart understanding of physiological and pathophysiological cardiac adaptation.

## 6 Conclusion

As the heart is constantly experiencing acute variations in mechanical load, for which it must adapt its activity to compensate, MEC and MMC are critical physiological responses. Little is known about the presence and important of these responses *in vivo* in the developing heart, in part due to current experimental limitations. The novel methodology presented here allows for rapid variation in the mechanical load of the larval zebrafish heart, while simultaneously imaging its functional effects, which may be used in future studies to better understand the role of MEC and MMC for acute adaptation during cardiac development.

## Data Availability

The raw data supporting the conclusion of this article will be made available by the authors, without undue reservation.
